# The Answer Lies in the Energy: How Simple Atomistic Molecular Dynamics Simulations May Hold the Key to Epitope Prediction on the Fully Glycosylated SARS-CoV-2 Spike Protein

**DOI:** 10.1021/acs.jpclett.0c02341

**Published:** 2020-09-04

**Authors:** Stefano A. Serapian, Filippo Marchetti, Alice Triveri, Giulia Morra, Massimiliano Meli, Elisabetta Moroni, Giuseppe A. Sautto, Andrea Rasola, Giorgio Colombo

**Affiliations:** † Department of Chemistry, University of Pavia, viale Taramelli 12, 27100 Pavia, Italy; ‡ Department of Chemistry, University of Milan, via Venezian 21, 20133 Milano, Italy; § SCITEC−CNR, via Mario Bianco 9, 20131 Milano, Italy; ∥ Center for Vaccines and Immunology, Department of Infectious Diseases, University of Georgia, 501 D. W. Brooks Drive, Athens, Georgia 30602, United States; ⊥ Dipartimento di Scienze Biomediche, Università di Padova, viale G. Colombo 3, 35131 Padova, Italy

## Abstract

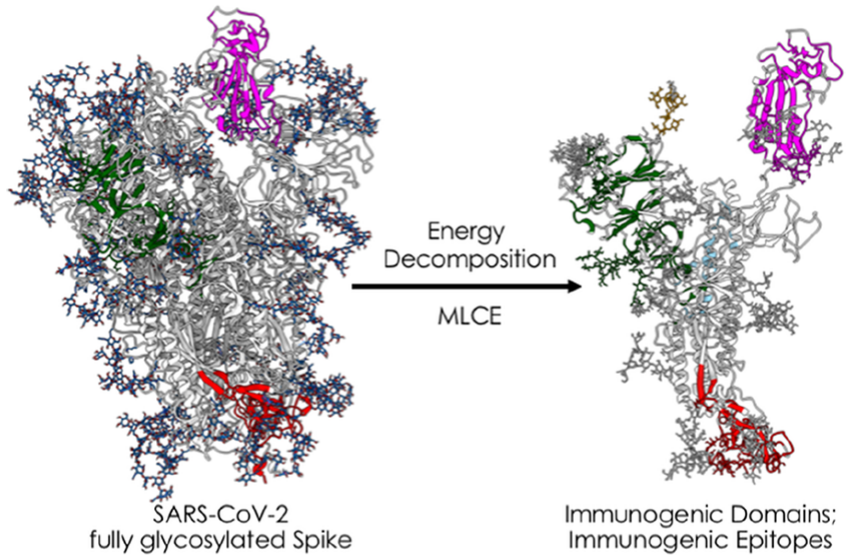

SARS-CoV-2 is a health threat with dire socioeconomical consequences. As the crucial mediator of infection, the viral glycosylated spike protein (S) has attracted the most attention and is at the center of efforts to develop therapeutics and diagnostics. Herein, we use an original decomposition approach to identify energetically uncoupled substructures as antibody binding sites on the fully glycosylated S. Crucially, all that is required are unbiased MD simulations; no prior knowledge of binding properties or ad hoc parameter combinations is needed. Our results are validated by experimentally confirmed structures of S in complex with anti- or nanobodies. We identify poorly coupled subdomains that are poised to host (several) epitopes and potentially involved in large functional conformational transitions. Moreover, we detect two distinct behaviors for glycans: those with stronger energetic coupling are structurally relevant and protect underlying peptidic epitopes, and those with weaker coupling could themselves be prone to antibody recognition.

The novel coronavirus SARS-CoV-2, the etiological agent of COVID-19 respiratory disease, has infected millions of people worldwide, causing more than 800 000 deaths (as of August 30, 2020) and extensive social and economic disruption. Given the pandemic status of the outbreak, social distancing measures cannot be sufficient any longer to contain it on a worldwide scale. This emergency calls for the development of strategies to rapidly identify pharmacological agents or vaccines as the only way to contain and combat the disease in order to restore normal social conditions. Indeed, a number of currently ongoing trials focus on developing vaccines (see, e.g., https://www.nytimes.com/interactive/2020/science/coronavirus-vaccine-tracker.html) or on repurposing drugs already developed for other disorders.^1−4^

SARS-CoV-2 is extraordinarily effective in exploiting the host’s protein machinery for replication and spreading. This is a characteristic that it shares with other members of the Coronaviridae family, all of which are characterized by a highly selective tropism that determines the onset of a variety of diseases in domestic and wild animals as well as in humans, including central nervous system affections, hepatitis, and respiratory syndromes.^5,6^ As was the case with its human predecessors SARS-CoV and MERS, the homotrimeric viral spike protein (S) (Figure 1) is the key player regulating cell entry, with the protein receptor angiotensin-converting enzyme 2 (ACE2) representing the host cell docking point in SARS-CoV-2 and SARS-CoV.^7,8^ The CoV S protein is then cleaved by a series of serine proteases, including trypsin, cathepsins, elastase, the host type 2 transmembrane serine protease (TMPRSS2), and plasmin, which promote virus entry into epithelial cells.^4^ In this context, it is important to underline that many vaccines under development for SARS-CoV-2 indeed focus on using recombinant forms of the S protein.

**Figure 1:**
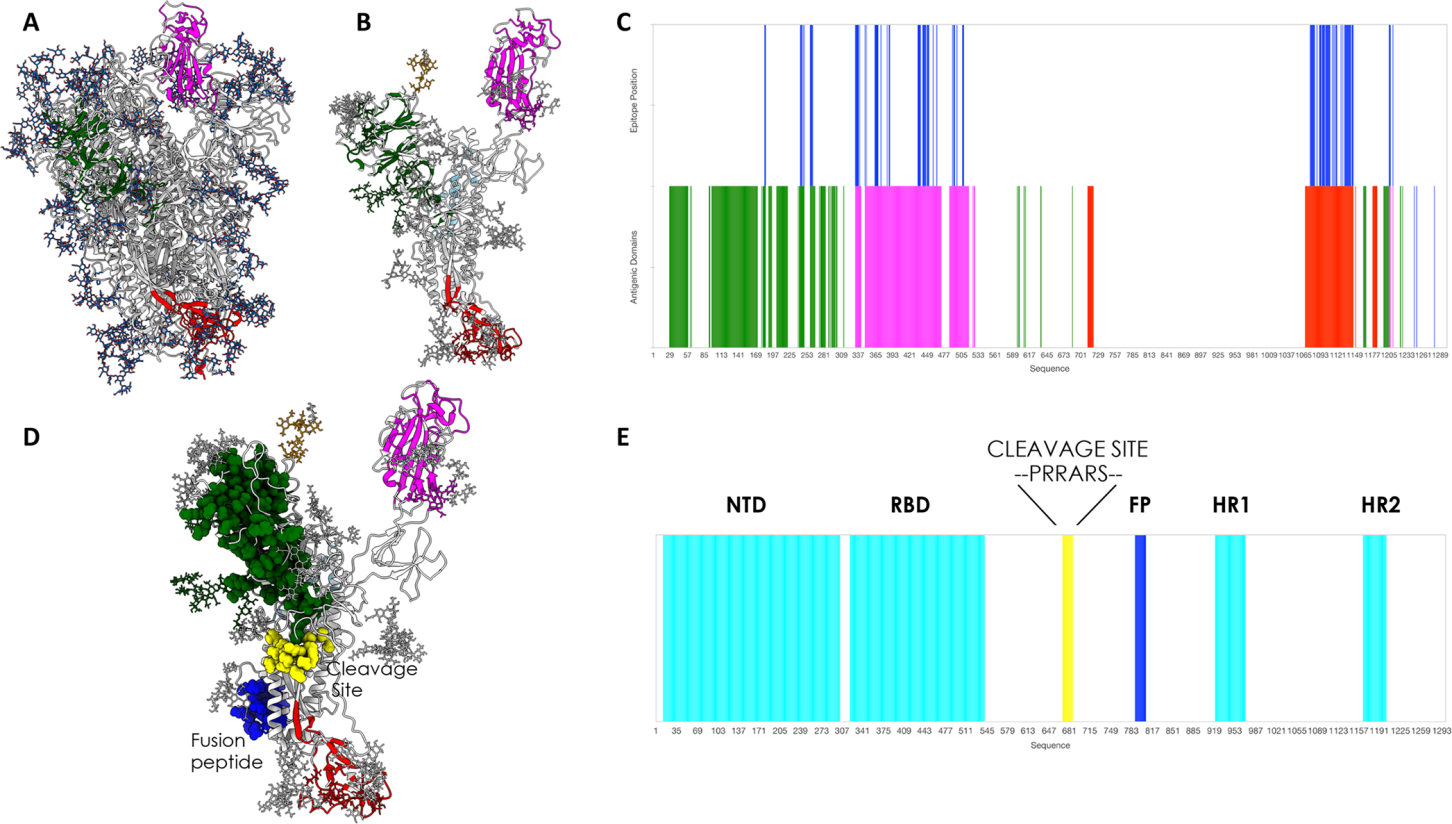
3D structure, glycosylation, and location of antigenic domains and epitopes on SARS-CoV-2 fully glycosylated spike protein. (A) Starting fully glycosylated spike protein trimer. The coating oligosaccharides are colored in dark blue. The predicted antigenic domains are colored on the structure of one protomer. (B) Isolated protomer with the most antigenic domains, detected via MLCE with the 15% cutoff, highlighted in colors: dark green for the antigenic part in the N-terminal domain, magenta for the part in the RBD, and dark red for the part in the C-terminal domain. Oligosaccharides that define or are part of antigenic domains are also colored. Oligosaccharides that have a structural role and show strong energetic coupling to the protein are depicted in white. (C) Predicted antigenic sequences projected on the sequence of the protein. The bottom line reports the sequences defined as antigenic domains, with the same color code as in (B). The top bar reports the location of peptidic epitopes identified with the most restrictive definition. (D) Physical interaction between the boundaries of the predicted antigenic domain in the N-terminal region and the cleavage site of S. This panel also shows the physical proximity of the predicted C-terminal uncoupled region with the fusion peptide. (E) Domain organization of the spike protein projected on the sequence. Numbering and domain definitions were obtained from UniProt (https://www.uniprot.org/uniprot/P0DTC2).

Recent cryogenic electron microscopy (cryo-EM) analyses allowed precise determination of the structure of the full-length spike protein in its trimeric form^9−11^ and the structural basis for the recognition of the spike protein’s receptor binding domain (RBD) (Figure 1) by the extracellular peptidase domain of ACE2.^7^ In parallel, computational studies have started to provide atomically detailed insights into S protein dynamics and the elaborate role of the diverse polysaccharide chains that decorate its surface in effectively shielding a large portion of it from the host.^12−14^ Computational approaches have also started to shed light on the determinants of binding to host cell receptors, studying in particular the interactions of the S protein with ACE2.^15−17^

This detailed dynamic and structural knowledge can set the stage for understanding the molecular bases of S protein recognition by the host’s immune system, providing information on which physicochemical determinants are required to elicit functional antibodies (Abs). Such understanding can then be exploited to design and engineer improved antigens based on S, for instance by identifying antigenic domains that can be expressed in isolation or short sequences (epitopes) that can be mimicked by synthetic peptides.^18−23^ This would be a crucial first step in the selection and optimization of candidate vaccines and therapeutic Abs (on top of those already in development) as well as in the development of additional serological diagnostic tools.

Even more significantly, knowledge acquired today about such recognition mechanisms could well mean that we are better prepared to tackle similar pandemics in the future by contrasting them more efficiently through the application of the same efficient and well-tested methods to new protein variants. More specifically, upon emergence of a new pathogen, generally portable computational methods could be advantageously exploited to rapidly identify and synthesize recombinant antigen- or peptide-based vaccines.

Here we analyze representative 3D conformations of the full-length trimeric S protein in its fully glycosylated form (Figure 1), extracted from atomistic molecular dynamics (MD) simulations provided by the Woods group,^13,24^ in order to predict immunogenic regions. To this end, a simple ab initio epitope prediction method that we previously developed for unmodified proteins^25−29^ is optimized and extended to cover glycoproteins. The method is based on the idea that Ab recognition sites (epitopes) may correspond to localized regions that exhibit only low-intensity energetic coupling with the rest of the structure. Otherwise put, putative interacting patches are hypothesized to be characterized by nonoptimized intramolecular interactions with the remainder of the protein. Actual binding to an external partner such as an Ab is expected to occur if favorable intermolecular interactions determine a lower free energy for the bound state than for the unbound state.^25,27,30^ Furthermore, minimal energetic coupling with the rest of the protein provides these subregions with greater conformational freedom to adapt to and be recognized by a binding partner^28,29^ as well as improved tolerance to mutations at minimal energetic expense without affecting the protein’s native organization and stability in a way that could be detrimental for the pathogen. All of these properties are indeed hallmarks of Ab-binding epitopes.

We show that our approach is indeed able to identify regions—also comprising carbohydrates—that recent structural immunology studies have shown to be effectively targeted by Abs. On the same basis, our method predicts several additional potential immunogenic regions (currently still unexplored) that can then be used to generate optimized antigens, either in the form of recombinant isolated domains or as synthetic peptide epitopes. Finally, our results help shed light on the mechanistic bases of the large conformational changes underpinning biologically relevant functions of the protein.

To the best of our knowledge, this approach is one of the first that permits the discovery of epitopes in the presence of glycosylation (an aspect that is often overlooked) starting only from an analysis of the physicochemical properties of the isolated antigen in solution. Importantly, the approach does not require any prior knowledge of Ab binding sites of related antigenic homologues and does not need to be trained/tuned with data sets or ad hoc combinations of information on sequences, structures, solvent-accessible surface area (SASA), or geometric descriptors. The procedure is thus immediately and fully portable to other antigens.

To reveal the regions of the S protein that could be involved in Ab binding, we employ a combination of the energy decomposition (ED) and matrix of low coupling energies (MLCE) methods, which we previously introduced and validated^25−27,31−39^ and discuss in full in Methods.

Starting from six combined 400 ns replicas of atomistic MD simulations of the fully glycosylated S protein in solution^13,40^ (built from PDB entry 6VSB(9)), we isolate a representative frame from each of the three most populated clusters. ED and MLCE analyses of protein energetics assess the interactions that each amino acid and glycan residue in S protomers establish with every other single residue in the same protomer. In particular, we compute the nonbonded part of the potential energy (van der Waals, electrostatic interactions, and solvent effects) implicitly via a molecular mechanics/generalized Born surface area (MM/GBSA) continuum solvation calculation,^41^ which for a protomer composed of *N* residues (including monosaccharide residues on glycans) gives a symmetric *N* × *N* interaction matrix **M**. Eigenvalue decomposition of **M** highlights the regions of strongest and weakest coupling. The map of pairwise energy couplings can then be filtered with topological information (namely, the residue–residue contact map) to identify localized networks of low-intensity coupling (i.e., clusters of spatially close residue pairs whose energetic coupling to the rest of the structure is weak and not energetically optimized through evolution).

In our model, when these fragments are located on or near the surface, contiguous in space and weakly coupled to the protein’s “stability core”, they represent potential interaction regions (i.e., epitopes).

Once interacting vicinal residue pairs (*i*, *j*) are identified by cross-comparison with the residue–residue contact map (vide supra and Methods), identification of poorly coupled regions representing potential epitopes proceeds as follows. Residue pairs are first ranked in order of increasing interaction intensity (from weakest to strongest). Two distinct sets of energetically decoupled regions are then mapped by applying two distinct cutoffs (“softness thresholds”) to the residue pair list: either from the first 15% or from the first 5% of the ranked pairs (i.e., the 5% or 15% of the residue pairs with the weakest energetic coupling). As a caveat, it is worth noting here that different S protomer conformations may provide slightly different results, as the interaction matrix is naturally dependent on the structural organization of the protein. Here we use the combination of predicted energetically uncoupled sequences as the proposed immunoreactive domains or substructures.

The less restrictive 15% cutoff subdivides the full-length, fully folded S protein into potentially immunoreactive domains (see Figure 1B,C and Methods).^26,30,32^ The goal is to uncover regions that may normally be hidden from recognition by Abs in the native protein structure but can be experimentally expressed as isolated domains. Highly reactive neutralizing epitopes may in fact be present only in specific but transient conformations that are not immediately evident in the static X-ray and EM models of the protein or are not accessible even to large-scale MD simulations. Presenting these (cryptic) regions for Ab binding through their isolated parent domains may prove more advantageous in developing new immunogens.^26,32^

The more stringent epitope definition (5% cutoff) narrows the focus on those (smaller) intradomain regions that could be directly involved in forming the interface with Abs, which can then be used to guide the engineering of optimized antigens in the form of synthetic epitope peptidomimetics. In this context, to be defined as epitopes, the energetically uncoupled regions must be at least six residues long.

Upon use of the larger cutoff value, a large cluster of energetically unoptimized residue pairs localize at the RBD, correctly identifying it as the most antigenic unit in the S protein’s “RBD up” protomer (Figure 1B,C, magenta-colored domain). Interestingly, when the lowest-energy coupled residue pairs are mapped onto the “up” RBD of all three 3D structures isolated from MD, there is a large overlap with regions recognized by Abs and nanobodies (as revealed by recent X-ray and cryo-EM structures). Importantly, for example, our calculation correctly identifies the binding region of the monoclonal Ab (mAb) CR3022^42^ (see PDB entry 6W41), known to target a cryptic epitope that is exposed only upon significant structural rearrangement of the protein^12^ (Figures 2 and 4).

**Figure 2:**
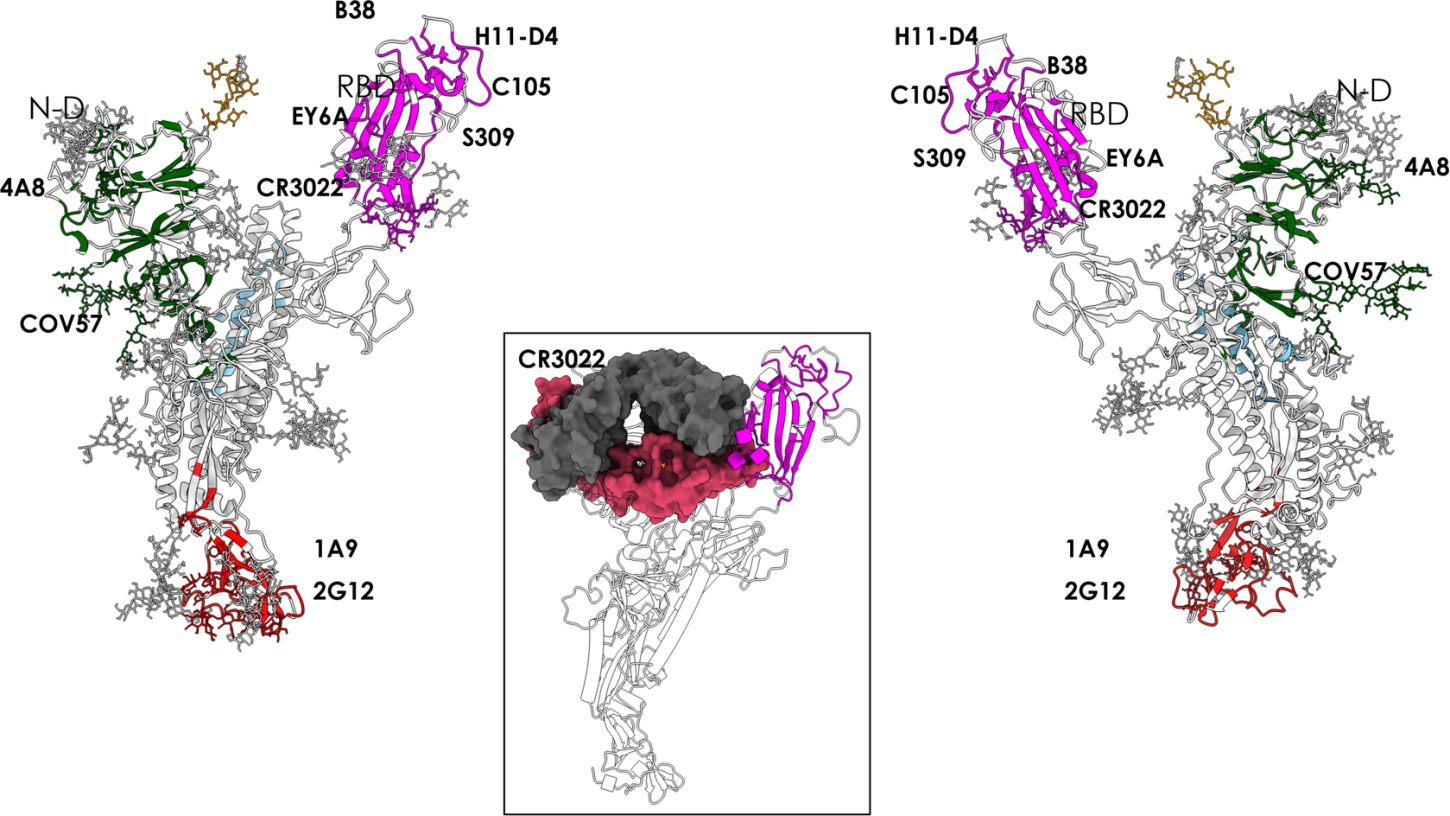
Antigenic domains and location of binding antibodies. Clusters of residues defining antigenic domains (dark green in the N-terminal domain, magenta in the RBD, red in the C-terminal region) and the positions of the various antibodies whose structures and interactions in complexes with the full length protein have been described are shown. The inset indicates the identification of the cryptic immunoreactive region that binds CR3022.

A second domain that is found to host a large network of nonoptimized interactions corresponds to the N-terminal domain (Figure 1B–D). The latter has been shown to bind the new antibody 4A8 (PDB entry 7C2L) in a paper that was published during the preparation of this Letter.^43^

A third region predicted to be highly antigenic coincides with the central/C-terminal part of the S1A domain. In a recent cryo-EM study of polyclonal antibodies binding to the S protein, this substructure was shown to be in the vicinity of the density for antigen-binding fragment(s) (Fab(s)) of COV57, a novel Ab whose neutralizing activity showed no correlation with that of RBD-targeting Abs^44^ (Figure 1B,D). We note here that MERS Ab 7D10 also binds in this region.^45^

Furthermore, MLCE identifies a potentially highly reactive region in the S2 domain of the protein, in the CD region. This domain contains the epitope recently found to engage with 1A9,^46^ an Ab that was recently shown to cross-react with S proteins of human, civet, and bat coronaviruses. This analysis also recognizes a potential antigenic region in a carbohydrate cluster located in the S2 domain of the protein. Intriguingly, recent findings indicate that an oligosaccharide-containing epitope centered around this predicted region is targeted by the glycan-dependent antibody HIV-1 bnAb 2G12^47^ (Figures 1 and 2).

Identification of energetically uncoupled domains also has mechanistic implications. Regions that are not involved in major intramolecular stabilization can be displaced from the biomolecule at minimal energetic cost, sustaining large-scale conformational changes that typically underpin its biological function. The boundary of the (uncoupled) N-terminal region (Figure 1, dark-green domain) lies in physical proximity to the furin-targeted motif RRAR, which is essential for preactivation of SARS-CoV-2 spike protein through proteolysis. Thus, the large uncoupled region of the N-terminal domain can synergize with (and favor, through domain displacement) cleavage of this motif, ultimately favoring detachment of the S1 domain and release of the S2 fusion machinery.^9−11,48^ Furthermore, the β-sheet at the initial boundary of the C-terminal domain in S2 (red domain in Figure 1) is in close physical proximity to the fusion peptide (Figure 1D,E). Here it would be reasonable to expect that exposure or conformational rearrangement of the C-terminal domain is favored by its nonoptimized interactions with the core of the S protein stalk and would in turn optimally expose the fusion peptide, favoring its integration with the host membrane.^48^

Overall, these findings support the validity of our approach in identifying protein domains that can be aptly used as highly reactive immunogens, as they are most likely to be targeted by a humoral immune response. Our analysis predicts that regions other than the S protein RBD may represent alternative targets for neutralization or functional perturbation of SARS-CoV-2. On the one hand, this may be important in view of the fact that the RBD can also be the target of non-neutralizing antibodies (e.g., CR3022^42^). Indeed, using cocktails of antibodies to target different regions of S has recently been proposed as a viable therapeutic option,^43^ and Ab cocktails have been successfully used in the treatment of other epidemics such as Ebola. In this context, the company Regeneron is pursuing a cocktail-type approach for SARS-CoV-2 that is already in clinical trials.^49^

Turning to our more stringent definition of epitope, based exclusively on the top 5% of the most weakly coupled residue pairs (5% cutoff), we next focus on those regions of the S antigen that can be involved in forming contacts with antibodies.

Importantly, one predicted conformational epitope with sequence (348)A-(352)A-(375)S-(434)IAWNS(438)-(442)DSKVGG(447)-(449)YNYL(452)-(459)S-(465)E-(491)PLQS(494)-(496)Q-(507)PYR(509) encompasses regions of the S protein in contact with the antibodies C105 (PDB entry 6XCN),^44^ S309 (PDB entries 6WPT and 6WPS),^50^ and AB23 (PDB entry 7BYR);^51^ with the nanobody H11-D4 (PDB entry 6Z43); and with a recently reported synthetic nanobody (PDB entry 7C8V) (Figure 3).

**Figure 3:**
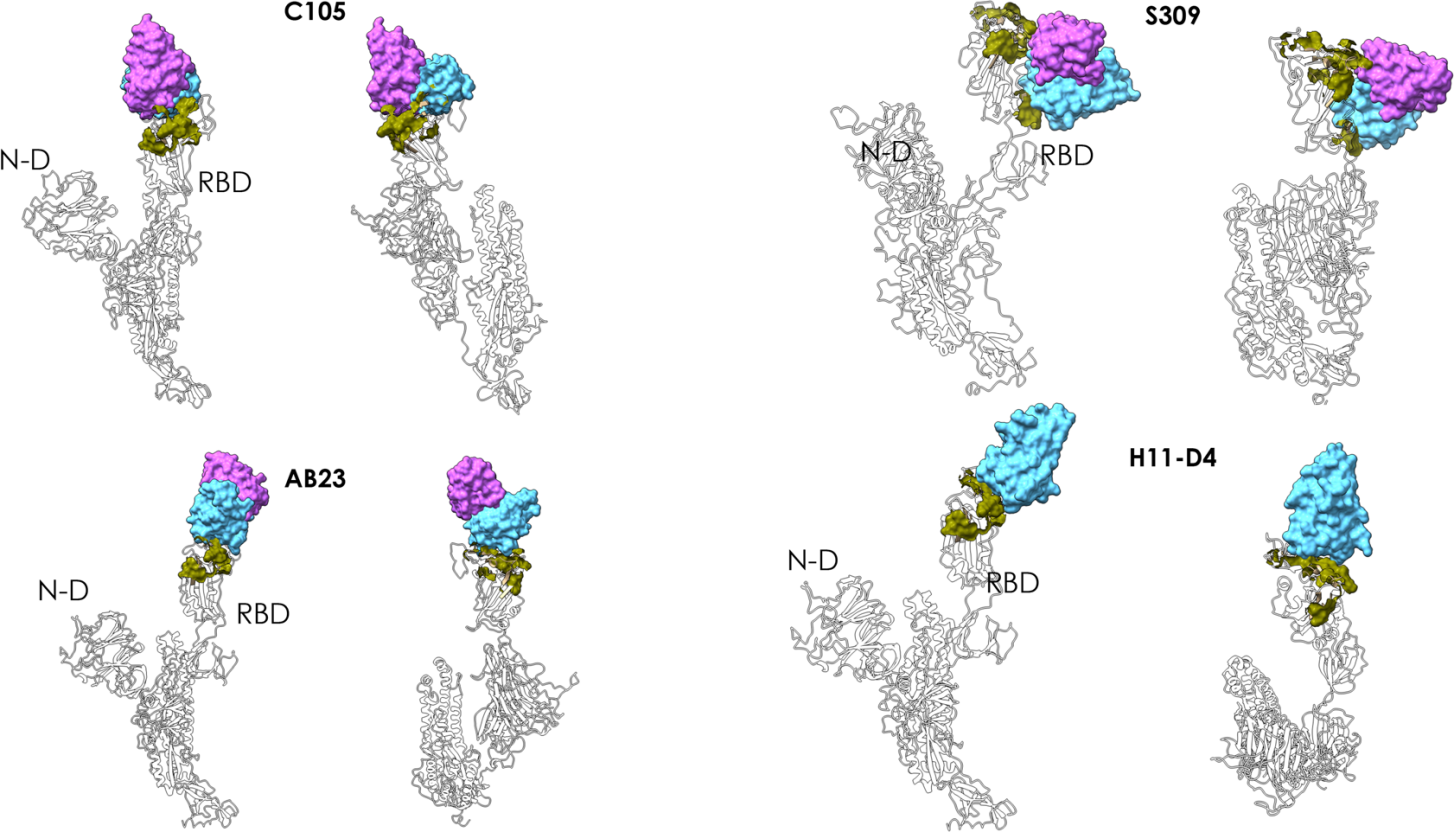
Peptidic epitopes predicted on the surface of the RBD using the restrictive definition of the antigenic region and comparison with known Ab complexes. The X-ray structures of the complexes between the various antibodies reported in the figure (C105, S309, AB23, and nanobody H11-D4) and the full length spike protein are superimposed on the structure of the protomer used here for prediction. The green surfaces indicate the location of MLCE epitope predictions. The Fabs of the antibodies and the nanobody are depicted as accessible surfaces in shades of blue.

Interestingly, an additional predicted patch comprising a set of decorating carbohydrates is correctly predicted to be part of the interface with antibody S309,^50^ with amino acid sequence (332)ITNLC(336)-(361)C and with the (N334*-*linked) fucosylated *N*-glycan chitobiose core (Manβ1–4GlcNAcβ1–4[Fucα1–6]GlcNAcβ-Asn).^52^ This predicted region sits notably close to the RBD interaction surface with ACE2.

Antibody EY6A (PDB entry 6ZDH) binds the RBD in the region of the cryptic epitope described by Wilson and collaborators^42^ (Figure 4). Importantly, our predicted patch (365)YSVLYN(370)-(384)PTKLN(388) covers a significant part of the epitope. Once again, it is worth remarking that identification of this potentially immunoreactive patch is simply and exclusively obtained from structural and energetic interaction data generated for a protomer of the glycosylated isolated S protein after unbiased MD simulations (see Methods).

**Figure 4:**
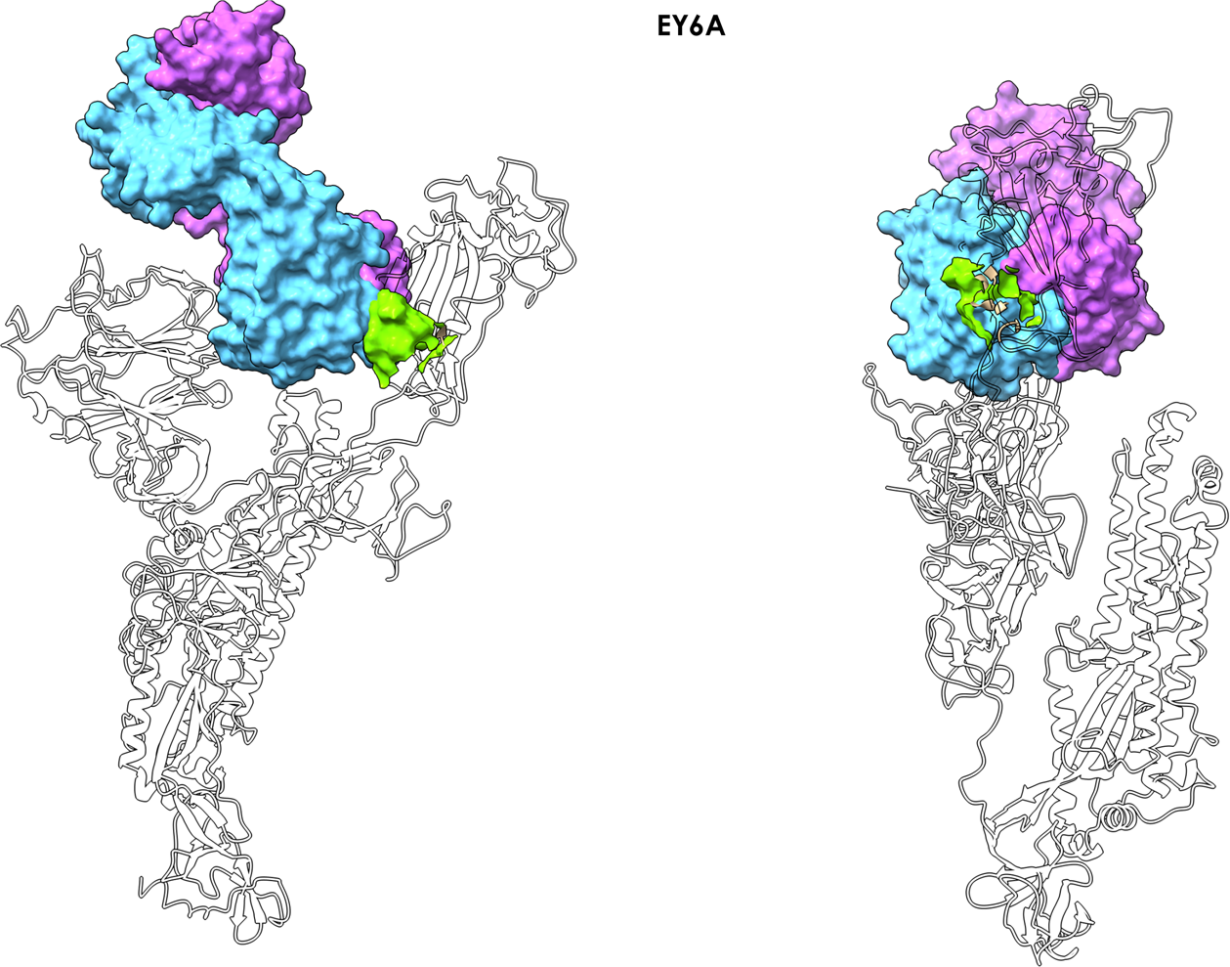
Antibody EY6A–spike complex. The figure shows how antibody EY6A (PDB entry 6ZDH) binds the RBD in the region of a cryptic epitope. The MLCE-predicted epitope region is shown in light green (lime) in two different orientations, indicating substantial contact formation with the antibody.

With the more restrictive epitope prediction cutoff we clearly identify a reactive area in the N-terminal domain of the Spike protein. The predicted patch (184)GN(185)-(242)LAL(244)-(246)R-(248)Y-(258)WTAGA(262) contains residues R246 and W258, which were described as central determinants for contact between the N-terminal domain and antibody 4A8^43^ (Figure 5).

**Figure 5:**
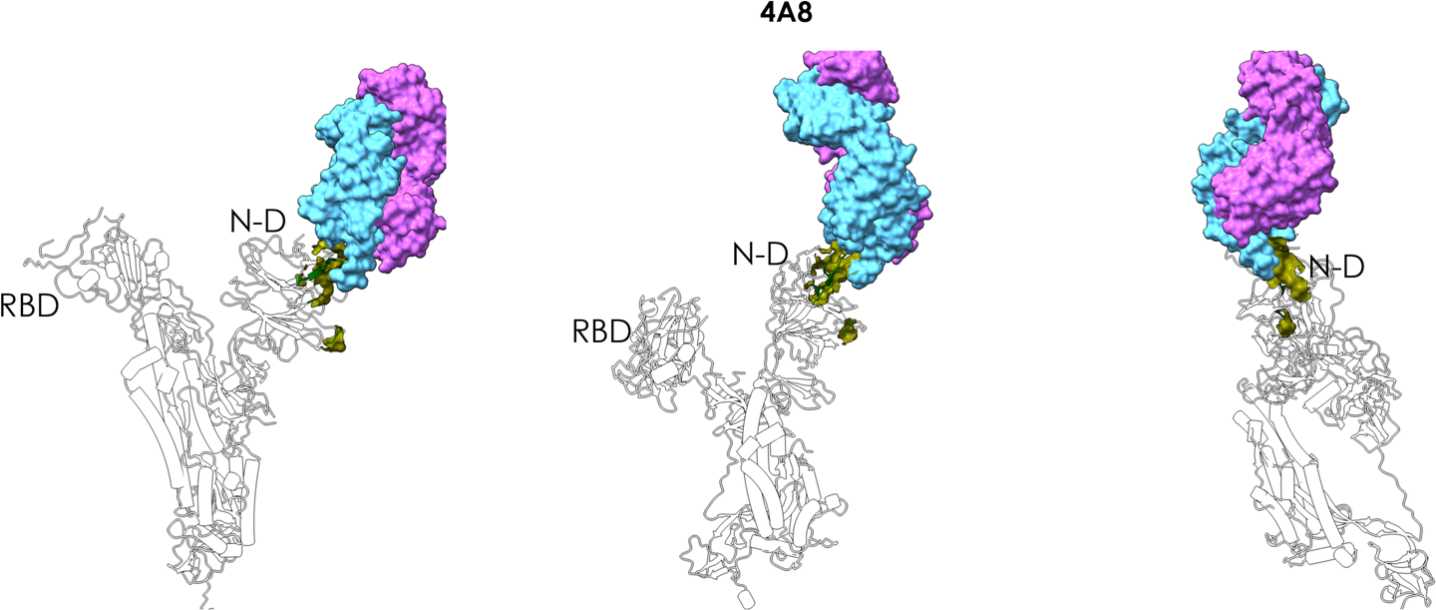
Antibody 4A8–spike complex. The figure shows how 4A8 binds the N-domain of spike, supporting the correct prediction of the epitope. The MLCE-predicted epitope region is shown in green in three different orientations, indicating substantial contact formation with the antibody. The Fab of the antibody is depicted as accessible surface in shades of blue.

Finally, the restrictive prediction identifies the sequence spanning residues 1076–1146, which includes amino acids 1111–1130, experimentally identified as the epitope for mAb 1A9.^46^ Specifically, our identified reactive sequence is the following: (1076)TTAPAICH(1083)-(1087)A-(1092)REG(1094)-(1096)FVSNGHWFVTQRN(1108)-(1112)P-(1114)I-(1116)T-(1118)DN(1119)-(1126)C-(1129)V-(1132)IVNNTVYDPLQPELD(1146).

In general, our approach is able to identify potential immunoreactive domains and epitopes of the spike protein on the basis of only structural and energetic information: our approach correctly predicts 20–80% of the amino acids engaged by mAbs in reported X-ray structures. As different (combinations of) antigen residues may be shared among different antibodies in a polyclonal response (such as the one taking place in the host organism), capturing even the minimal sequence endowed with potential immunoreactivity can aptly represent a useful step toward designing molecules that can elicit Abs capable of interfering with viral entry or replication.

In this framework, sequences predicted to be reactive using the restrictive epitope definition (5% cutoff) can be used to generate optimized antigens in the form of synthetic peptide epitopes. Engineering such epitopes would entail the synthesis of conformationally preorganized peptidomimetics of the “natural” reactive regions—with intra- and extracellular stability enhanced through, e.g, a combination of natural and non-natural amino acids—that could reproduce the main structural and energetic conditions required to elicit a humoral immune response and thus constitute candidates for vaccine development. Furthermore, reactive peptides thus identified may be suitable for use as baits in serologic diagnostic applications (e.g., in ELISA assays and microarrays) to capture and detect not only circulating antibodies that are expressed in response to SARS-CoV-2 infections but also those that are endowed with neutralization activity, thus potentially predicting the infection outcome. As a further application, these peptide-based baits can represent a useful tool for isolating new mAbs and screening small molecules for drug development.

One of the most significant aspects of our approach is that the S protein’s entire glycan shield is explicitly taken into account in the prediction of the immunoreactive regions. Indeed, the various oligosaccharide chains appear to behave differently (see the differential coloring of oligosaccharide chains in Figure 1). In light of their stronger energetic coupling to other areas of the protein, some of the glycans are not recognized as epitopes and thus form an integral part of the stabilizing intramolecular interaction network of S (white chains in Figure 1B); on the other hand, MLCE also identifies a second subset of poorly coupled oligosaccharides as potentially reactive epitopes (or parts thereof) (colored oligosaccharide chains in Figure 1B; carbohydrate cluster in S2 targeted by the glycan-dependent antibody HIV-1 bnAb 2G12, see Figures 1B and 2), highlighting potential vulnerable spots in the glycan shield that could be exploited to design novel immunoreagents and vaccine candidates.

The portion of the glycan shield that falls within the former category thus mainly serves to *protect* the protein from recognition by antibodies and consequently enhances viral infectiousness as well as providing extra structural support. Two such glycans are further exemplified in Figure 6. The first is the entire oligosaccharide fragment bound to N234 (Figure 6A), which was recognized by Amaro and co-workers as being crucial in “propping up” the RBD.^12^ Experimental deletion of *N*-glycans at this position by way of a mutation to Ala significantly modifies the conformational landscape of the protein’s RBD.^53^ The second is the portion of the N165-linked glycan whose subunits are rendered in yellow (Figure 6B). Consistent with experimental studies indicating that N165-linked oligosaccharides act as structural modulators,^53^ we also find that the portion in question is *not* identified as a potential epitope and consequently is involved in diverting antibodies from targeting the region around N165, thus preserving control of the S protein’s structural dynamics. Reflecting the multifaceted roles of the glycan shield, the remaining part of the N165-linked glycan (Figure 6B, orange) appears instead to belong to the category of glycans that *are* potentially able to act as epitopes since, unlike the part in yellow, we do detect it to be decoupled from the rest of the protomer.

**Figure 6:**
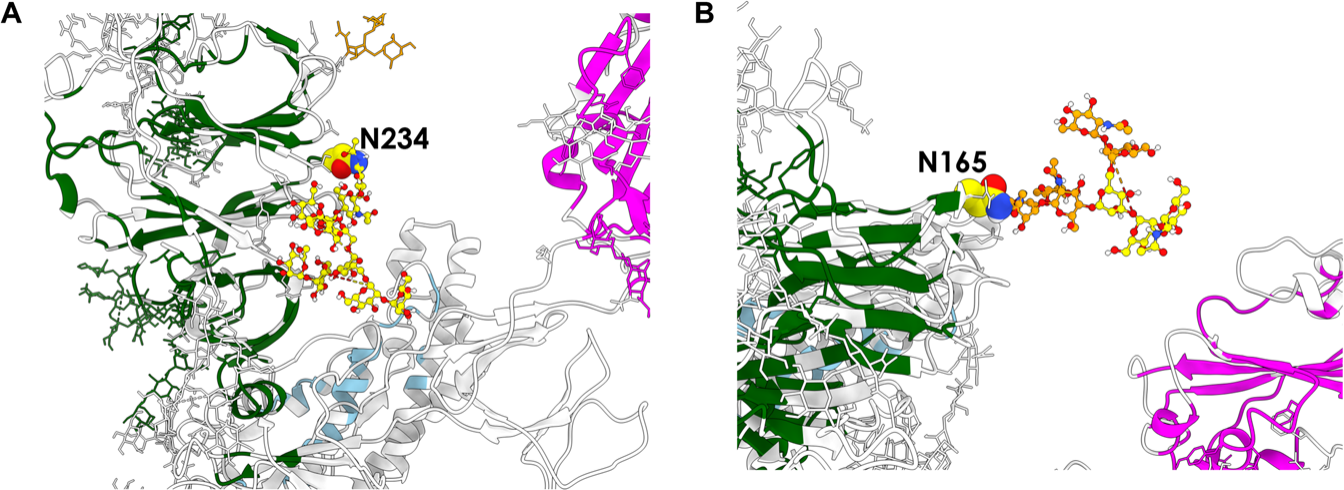
Glycans with different roles on spike protein. (A) The glycan chain attached to N234 is predicted to be part of the networks of stabilizing interactions within the protein. (B) The glycan chain attached to N165 is predicted to play a double role, a stabilizing one (yellow units) and an immunoreactive one (orange units).

lt is particularly significant to underline that MLCE, whose physical basis is to identify nonoptimized interaction networks, detects peptidic epitopes even when they are in proximity to (optimized, nonimmunogenic) shielding carbohydrates. In light of this, it is reasonable to suggest that the protective effect of these particular carbohydrates may be circumvented and neutralized by exposing the underlying peptidic substructures. Furthermore, information on oligosaccharides identified as epitope constituents can be exploited to design glycomimics or glycosylated peptides as synthetic epitopes.

The latter aspect is indeed particularly relevant: small synthetic molecules that mimic antigenic determinants (and effectively act as their minimal surrogates) offer enticing opportunities to develop immunoreagents with superior characteristics in terms of ease of handling, reproducibility of batch-to-batch production, ease of purification, sustainable cost, and better stability under a variety of conditions. Furthermore, production of these molecules greatly reduces the risk of cross-reactivity with any copurified antigens, which is instead rife when dealing with recombinant proteins. In contrast to smaller peptides or sugar-decorated peptidomimetics, a full-length recombinant antigenic protein (or any protein-based detection device) would typically require more stringent conditions (e.g., in terms of temperature and humidity) for storage, transport, and management in order to preserve the protein in its properly folded active form. The same would be true for other vaccinal solutions such as deactivated pathogens.

Overall, our work confirms how simple and transparent structural and physicochemical understanding of the molecule that is the key player in SARS-CoV-2 viral infection can be harnessed to guide the prediction of (in some cases experimentally confirmed) regions that are involved in immune recognition and to understand its molecular bases. Agreement with experiment confirms that knowledge generated in the process has the potential to be translated into new molecules for vaccine and diagnostic development. In this context, we have also identified potentially reactive regions in the S protein stalk that are currently under experimental synthesis and testing.

Furthermore, potential functional implications offered by the approach are illustrated by the fact that domains/regions that are relevant for the protein’s biological activation are naturally identified. This renders the approach well-suited to identify subtle functional variations in possible mutants of the S proteins that are expected to emerge as a result of further viral diffusion and host adaptation. Finally, the possibility of accurately partitioning such a complex system in functional subunits could aptly be exploited in the parametrization of coarse-grained models to simulate the system at longer time scales.

This kind of structure-based computational approach can clearly expand the scope of simple structural analysis and molecular simulations. In applicative terms, generation of synthetic libraries based on predicted/identified epitopes (with possible addition of sugars) would definitely boost selection and screening of antigens for vaccine development.

## Methods

Coordinates of the fully glycosylated SARS-CoV-2 S protein’s “RBD up” protomer featured in this work originated from MD simulations by Woods and co-workers^13^ based on PDB entry 6VSB. Throughout this work, we retained exactly the same force field parameters used by Woods et al. in their MD simulations: all residues except glycosylated asparagines were treated using the ff14SB force field,^54^ whereas glycans and glycosylated asparagines were modeled using the GLYCAM_06j force field.^52^

Clustering was based on the root-mean-square deviation of Cα atoms of the RBD domain in the “RBD up” protomer and performed with the *cpptraj* utility in AmberTools (version 17)^55^ after all six independent MD replicas^13^ were concatenated and then aligned with the “autoimage” command. The chosen method was the hierarchical agglomerative algorithm^56^ with ε = 0.5. From each of the three most populated clusters, we isolated one representative frame, from which we retained the “RBD up” protomer and its glycans and again used *cpptraj* to discard all solvent molecules and ions and the two “RBD down” protomers. All subsequent calculations on these three “RBD up” protomer models are listed chronologically in what follows.

A 200-step minimization of each of the three protomer models was carried out using the default procedure (i.e., steepest descent for 10 steps; then conjugate gradient) implemented in the MD engine *sander* in the AMBER software package (version 18).^55^ The protomers were minimized using the generalized Born implicit solvent model as parametrized by Onufriev et al.,^57^ with a universal 12.0 Å cutoff applied in the calculation of Lennard-Jones and Coulomb interactions (neither of which were calculated beyond this limit). For this stage, the concentration of (implicit) mobile counterions in the GB model was set to 0.1 M, and the SASA was computed according to the linear combinations of pairwise overlaps (LCPO) method.^58^

MM/GBSA calculations^41^ were performed on each of the three minimized “RBD up” protomers using the dedicated *mm_pbsa.pl* utility in AmberTools (version 17). The purpose of these calculations was to obtain a breakdown of nonbonded energy interactions (i.e., electrostatic, van der Waals, and implicit solvation contributions and, in this case, 1–4 interactions) between every possible pair of residues in the protomer (amino acids and monosaccharides alike); for a protomer composed of *N* residues, this leads to a symmetric *N* × *N* interaction matrix **M**.^59,60^

The implicit GB solvation model used in these calculations was identical to the one used in the preceding minimization step (vide supra), except that the implicit ion concentration was set to 0.0 M and the SASA was computed with the ICOSA method (based on icosahedra around each atom that are progressively refined to spheres).

The elements *M*_*ij*_ of the symmetric interaction matrix **M** obtained from separate MM/GBSA calculations (vide supra) on each of the three S protein protomer models under study (with *N* residues) can be expressed in terms of the eigenvalues and eigenvectors of **M** as
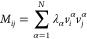
where λ_α_ is the αth eigenvalue and *v*_*i*_^α^ is the *i*th component of the corresponding eigenvector.

It was previously shown in a number of cases that eigenvector **v**^1^ (also called the *first eigenvector*), which is associated with the lowest eigenvalue λ_1_, allows the identification of most of the crucial amino acids necessary for the stabilization of a protein fold and consequently those amino acids that are minimally coupled to such a core. The latter were shown to correspond to potential interaction regions.

In the case of multidomain proteins such as S, the first eigenvector is not sufficient, and additional eigenvectors are needed to capture the essential interactions for folding/stability and binding. The interaction matrix **M** was thus decomposed instead via the alternative approach developed by Genoni et al.^30^ In that scenario, the aim is to select the smallest set of *N*_e_ eigenvectors that cover the largest part of residues (i.e., components) with the minimum redundancy under the assumptions that (a) for each domain there should exist only one associated eigenvector recapitulating its most significant interactions; (b) each “domain eigenvector” has a block structure, whereby its significant components correspond to the residues belonging to the identified domain; and (c) combination of all of the significant blocks covers all of the residues in the protein. Matrix **M** can thus be reformulated as a simplified matrix **M̃** with elements *M̃*_*ij*_ given by
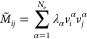
where this time the sum runs over the *N*_e_ essential eigenvectors instead of *N* residues. As detailed by Genoni et al.,^30^ the essential folding matrix **M̃** is subsequently further filtered through a symbolization process to emphasize the significant nonbonded interactions, yielding the matrix **M̃**^S^, which is finally subjected to a proper clustering procedure leading to domain identification. The final simplified matrix **M̃**^S^ resulting from domain decomposition thus reports only those residue pairs in the protomer that exhibit the strongest and weakest energetic interactions.

Final epitope predictions were made using the MLCE method, in which analysis of a given protein’s energetic properties is combined with that of its structural determinants.^25,27^ This approach allows the identification of nonoptimized contiguous regions on the protein surface that are deemed to have minimal coupling energies with the rest of the structure and thus have a greater propensity for recognition by Abs or other binding partners.

The MLCE procedure entails cross-comparison of the simplified pairwise residue–residue energy interaction matrix **M̃**^S^ resulting from domain decomposition (vide supra) with a pairwise residue–residue contact matrix **C**. The latter matrix considers a pair of residues to be spatially contiguous (i.e., “in contact”) if they are closer than an arbitrary 6.0 Å threshold; contact distances are measured between Cβ atoms in the case of non-glycine amino acid residues, H atoms in the case of glycine residues, and C1 atoms in the case of glycan residues.

The Hadamard product of the two matrices **M̃**^S^ and **C** yields the matrix of the local pairwise coupling energies, **MLCE**, whose elements are given by
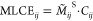
Deriving the MLCE matrix allows spatially contiguous residue pairs to be ranked with respect to the strengths of their energetic interactions (weakest to strongest). Selection of proximal pairs showing the weakest coupling with the rest of the protein ultimately defines putative epitopes; two distinct selections were carried out on the basis of two possible weakness (softness) cutoffs (5% or 15%), corresponding to the top 5% or 15% spatially contiguous residue pairs with the lowest-energy interactions.

## Supporting Information Available

Document with instructions on the structure–prediction files uploaded (PDF)
Machine-readable projection of the MLCE matrix onto the spike protein (ZIP)
The code to perform the analysis (ZIP)

## Notes

The code is also is available on GitHub at the following address: https://github.com/colombolab/MLCE.
