# Supplementary material for: The Answer Lies in the Energy: How Simple Atomistic Molecular Dynamics Simulations May Hold the Key to Epitope Prediction on the Fully Glycosylated SARS-CoV-2 Spike Protein
Source: J Phys Chem Lett. 2020 Sep 4;11(19):8084–93. doi: 10.1021/acs.jpclett.0c02341 (PMC7491317; doi:10.1021/acs.jpclett.0c02341)
Supplement: Supplementary file 1 — jz0c02341_si_001.pdf [file jz0c02341_si_001.pdf]

SUPPORTING INFORMATION

The answer lies in the energy: how simple atomistic molecular dynamics simulations may hold the key to epitope prediction on the fully glycosylated SARS-CoV-2 spike protein

Stefano A. Serapian,[†,@] Filippo Marchetti,[†,@,=] Alice Triveri,[†] Giulia Morra,[‡] Massimiliano Meli,[‡] Elisabetta Moroni,[‡] Giuseppe A. Sautto,[#] Andrea Rasola,[§] and Giorgio Colombo*[,†,‡]

[†]University of Pavia, Department of Chemistry, viale Taramelli 12, 27100 Pavia (Italy)

[‡]SCITEC-CNR, via Mario Bianco 9, 20131 Milano (Italy)

[#]Center for Vaccines and Immunology, Department of Infectious Diseases, University of Georgia, 501 D.W. Brooks Drive, 30602 Athens - GA (USA)

[§]Dipartimento di Scienze Biomediche, Università di Padova, viale G. Colombo 3, 35131 Padova (Italy)

[=]University of Milan, Department of Chemistry, via Venezian 21, 20133 Milano (Italy)

[@]These authors contributed equally.

Corresponding Author

* E-mail: g.colombo@unipv.it

Machine-Readable Projection of the MLCE Matrix onto the Spike Protein

$N \times N$ pairwise matrices of local coupling energies ($MLCE_{ij}$s; see main text) for clustering-derived models 1, 2, and 3 of protomer A (N = 1260 residues) have been collapsed to one dimension by summing, for each of the 1260 peptide and glycan residues, the MLCE contribution of all other 1259 residues (i.e., summation by columns). This leads to loss of (two-dimensional) pairwise information but generates a simplified one-dimensional vector, with one "generic" cumulative coupling value for each residue towards the rest of the protomer, that can be directly projected onto each model.

In the compressed archive (`MLCE_visualization.zip`), we provide a *PyMOL* session (`MLCE_visualization.pse`; version 2.3 and above) showing one-dimensional coupling vectors for models 1, 2, and 3 of protomer A projected onto their respective models on a residue-by-residue basis.

In these representations, we have opted for a symmetric blue-white-red color scale (cf. `mincol` to `maxcol` in the nomenclature), despite negative values (cf. `minmlce`) typically being one to two orders of magnitude greater than positive values (`maxmlce`). For readers wishing to generate their own visualization, in the same archive we also provide individual PDB files of the three models with per-residue one-dimensional MLCE values included in the B-factor column. In addition, we provide the original 1260 × 1260 $MLCE_{ij}$ matrix generated for each model by our code.

Code to Perform the Analysis

The most up-to-date version of the (*Python*) code required for the analysis (i.e., at the time of publication) is provided as a compressed archive (`MLCE.zip`). The bulk of the code was originally developed by Claudio Peri and Riccardo Capelli. A "README" file in the main directory lists known bugs and issues; provides information on the code's current version and how to correctly reference

it; and points to a manual with further information and installation instructions. The code is also available at https://github.com/colombolab/MLCE.
