# Supplementary material for: The Answer Lies in the Energy: How Simple Atomistic Molecular Dynamics Simulations May Hold the Key to Epitope Prediction on the Fully Glycosylated SARS-CoV-2 Spike Protein
Source: J Phys Chem Lett. 2020 Sep 4;11(19):8084–93. doi: 10.1021/acs.jpclett.0c02341 (PMC7491317; doi:10.1021/acs.jpclett.0c02341)
Supplement: Supplementary file 3 — jz0c02341_si_003.zip [file jz0c02341_si_003.zip › rebelot-1.2.31 2/doc/manual.pdf]

R.Capelli, C.Peri, G.Colombo

REBELOT Reference Manual

v.1.2.2

February 2017

Contents

1 Introduction **3**

2 Installation **5**

3 Usage **9**

3.1 REBELOT.py 9

3.1.1 Single frame mode (-m s) 11

3.1.2 Multiframe mode (-m m) 12

3.1.3 Cluster mode (-m c) 13

3.1.4 BEPPE mode (-m b) 14

3.2 pyBEPPE.py 15

Chapter 1

Introduction

REBELOT (Renewed and Extended BEppe Layer Over Trajectories) is a workflow script devoted to the study of the energetic footprint of proteins, designed to study the determinants of protein stability as well as the location of putative immunogenic epitopes.

The workflow is composed by two different executables:

- `REBELOT.py`, which contains the entire workflow,
- `pyBEPPE.py`, which perform the (optional) calculations to obtain the position of immunogenic epitopes.

In this manual, chapter 2 explains the installation process and the requirements for REBELOT workflow, chapter 3 illustrates the 3 different analysis methods.

Chapter 2

Installation

REBELOT is a Python2 script that perform calculations using non-standard libraries and external programs. It is designed to work on UNIX-based systems (it is tested on OS X and Linux).
REBELOT needs some packages to work

- Python 2.7+
- Numpy 1.8+

Furthermore, the external programs needed are

- AMBERTools 15/16
- gnuplot 4.4+

The AMBER MD package is used to perform the minimization on the structure (via the `sander` executable) and to compute the MM-GBSA per-residue energy decomposition (using the old `mm_pbsa.pl` routine, with experimental support for the new python routine, `MMPBSA.py`). You can also perform multicore structure minimization using `sander.MPI`, as explained below.
Gnuplot is used to plot the reconstructed energy matrix of the analyzed protein.

Once you have the gunzipped package `rebelot_1.2.2.tar.gz`, put it in the destination folder (e.g. `/home/rebelot/`)

```
$ mv rebelot_1.2.2.tar.gz /home/rebelot/.
```

and extract it

```
$ cd /home/rebelot
$ tar xvf rebelot_1.2.2.tar.gz
```

Now you have to open the script `REBELOT.py`, located in the `bin` directory and set the proper position of your python2 executable, just after the `#!` at line 1

```
#!/path/to/your/python
```

You can find the location of python executable on your machine using the command `which`

```
$ which python
/path/to/your/python
```

And do the same for the `pyBEPPE.py` script, also in the `bin` directory.
WARNING: check that your `python` executable is a python2 and **not** a python3 executable.
You have also to modify the path to retrieve AMBER-related executables:

- `tleap`;
- `mm_pbsa.pl`;
- `MMPBSA.py`;
- `sander`;
- `sander.MPI` (optional, for multicore minimization);
- `mpirun` (optional, for multicore minimization);

which are at lines 58-63 of the code.

Depending on the AmberTools version that you are using, you have to select the input files for the workflow:

- AmberTools 14/15:

```
54  'leaprc_bin': REB_PATH + '/data/leaprc.ff14SB.v14'
55  'minin_rc'  : REB_PATH + '/data/min.in'
```

- Ambertools 16:

```
54  'leaprc_bin': REB_PATH + '/data/leaprc.ff14SB.v16'
55  'minin_rc'  : REB_PATH + '/data/min16.in'
```

This choice is needed for changes in the force field (row 54) and in output format for restart files (row 55) in different AmberTools version.

Next, set the `PATH` environment variable to detect the executables

```
$ export PATH=$PATH:/home/rebelot/bin      #(bash, zsh,...)
$ setenv PATH $PATH:/home/rebelot/bin      #(csh, tcsh,...)
```

Finally REBELOT is ready to work. To test it, simply try

```
$ REBELOT.py -h
```

And check if the usage message is printed without errors (usually related to problem in python libraries).

Chapter 3

Usage

REBELOT is composed by two different programs, as we said in chapter 1: `REBELOT.py` and `pyBEPPE.py`.
`REBELOT.py` is the script that contains the entire workflow, which begins from a reference structure (or a small trajectory) in protein data bank format (.pdb) and returns the energy profile (sections 3.1.1, 3.1.2, and 3.1.3) or the prediction of the epitope(s) position(s) (section 3.1.4).

`pyBEPPE.py` is the part of the workflow devoted to the computation of the binding zones of the protein from the per-residue energy matrix. It can be used alone using a `REBELOT.py` output (`enematrix.dat`) to make one *a posteriori* fix of the sensitivity/specificity threshold for the epitope mapping.

3.1 REBELOT.py

`REBELOT.py` can work in three different modes:

- *Single frame* mode (`-m s`)
- *Multi frame* mode (`-m m`)
- *Cluster* mode (`-m c`)
- *BEPPE* mode (`-m b`)

The output of the program is inserted in a directory named `REBELOT` in the root directory; if it already exists, the script move the old directory to `REBELOT.$time`, there `$time` is the POSIX time when the old REBELOT instance was launched. After the directory move, a new `REBELOT` directory is created.

There is a common part between all the 3 modalities. `REBELOT.py` at the beginning reads the pdb structure of the system and using LEaP from AMBERTools[**?**] creates a reference structure (saved as `snapshot.AMBER.pdb`) and perform a short minimization in implicit solvent (GBSA) using the sander module. The resulting structure undergo to a MM-GBSA per-residue energy decomposition using mm_pbsa.pl module[1]. There is the possibility to use an existing GBSA matrix or a coevolutionary potential matrix[**?**, **?**] for the single frame analysis, skipping the minimization and the GBSA calculation.

The energy decomposition for a protein formed by N residues returns a $N \times N$ matrix which contains the mutual non-bonded (electrostatic and van der Waals) interactions between amino acids. The matrix (called M_{ij}) can thus be diagonalized and reconstructed using the resulting eigenvalues and eigenvectors

$$M_{ij} = \sum_{\alpha=1}^{N} \lambda_\alpha v_i^\alpha v_j^\alpha$$

Where λ_α is the α-th eigenvalue and μ_i^α are the i-th component of the corresponding eigenvector. Sorting the eigenvector from the most negative to the most positive, we can assume [**?**, **?**] that the first eigenvector labeled in this way contains most of the relevant information regarding the stabilization of the system. In practice

$$\sum_{\alpha=1}^{N} \lambda_\alpha v_i^\alpha v_j^\alpha \simeq \lambda_1 v_i^1 v_j^1 \qquad \longrightarrow \qquad M_{ij} \simeq \tilde{M}_{ij} = \lambda_1 v_i^1 v_j^1$$

In this way we have removed most of the noise of the weaker interaction intensities from the energy matrix.

An alternative way to rebuild the interaction matrix from the diagonalization is to select a subset of eigenvalues/eigenvectors as shown in [**?**]. This could be useful when a single eigenvectors does not describe properly all the structure (e.g. multidomain proteins). In practice

$$M_{ij} \simeq \tilde{M}_{ij} = \sum_{\alpha=1}^{M} \lambda_\alpha v_i^\alpha v_j^\alpha \qquad (M < N)$$

The subset of eigenvector is chosen in this way:

1. A threshold of significance for the eigenvectors component is set as the median of the absolute value of all the eigenvectors components. If the absolute

[1] There is also an unsupported flag, `--py`, that uses the python module MMPBSA.py instead. However, the results in the majority of cases are very different from the perl module and though this option is considered as experimental.

value of a component is larger than this threshold, that component is considered as significant;

2. The first eigenvector is pick;

3. All the eigenvectors are parsed, looking for the one which adds most information to the set of selected eigenvectors (e.g. which have the larger number of new significant components). If two eigenvectors add the same amount of information, the one with the most negative eigenvalue is selected.

4. In order to be inserted in the set, the eigenvector has to add an amount of information (defined as number of new components divided by the number of residues) greater than 0.01.

5. If at least 50% of the residues are covered by almost 3 eigenvectors, the new eigenvector is rejected and the selection is stopped. Otherwise, the eigenvector is added to the set and a new eigenvector has to be selected (step 3).

This decomposition technique (called domain decomposition) can be performed in all the REBELOT modes, using the flag `-d`.

3.1.1 Single frame mode (-m s)

In single frame mode, REBELOT works with the following syntax:

```
$ REBELOT.py -m s [-d] -f protein.pdb [--minrange aa1
      --maxrange aa2] [--matrix matrix.dat | --coevo
      coevo.dat] [--mpi np]
```

where the options refer to

Option	Description
`-d`	Activate domain decomposition
`-f filename`	Structure file
`--matrix filename`	(opt.) Raw interaction matrix
`--coevo filename`	(opt.) Coevolutionary potential matrix
`--minrange aa`	(opt.) First amino acid for decomposition
`--maxrange aa`	(opt.) Last amino acid for decomposition
`--mpi np`	(opt.) Number of threads for minimization

REBELOT in single frame mode generates some output files:

- `ele-vdw.dat` which contains the original matrix M_{ij} (if `--matrix` or `--coevo` options are not enabled)

- EIGENVAL.txt and EIGENVECT.txt, which contain the eigenvalue and the eigenvectors from the diagonalization of M_{ij};
- enematrix.dat, which contains the reconstructed energy matrix $\tilde{M}_{ij}$ and its plot enematrix.png;
- snapshot.AMBER.pdb, which is the structure parsed and possibly repaired by tleap;
- enedist.dat, which contains the distribution of interaction energy vs. the residue position, and the plot enedist.png
- REBELOT.log, which contains useful informations about the run, and all the output given by any part of the workflow.

There are also other file (e.g. input files created for sander, mm_pbsa.pl and their output).

3.1.2 Multiframe mode (-m m)

REBELOT can analyze small trajectories of a system. Having n frames in a trajectory, REBELOT consider every frame obtaining n matrices M_{ij}. After the analysis, it computes the average matrix $\langle M_{ij}\rangle$ and diagonalize it, obtaining the eigenvalues and eigenvectors that can be used to reconstruct the matrix $\tilde{M}_{ij}$. Obviously, the computation time scales with the number of frame contained in the trajectory.
In multiple frame mode, REBELOT works with the following syntax:

```
$ REBELOT.py -m m [-d] -f trajectory.pdb [--minrange aa1
      --maxrange aa2] [--mpi np]
```

where the options refer to

Option	Description
-d	Activate domain decomposition
-f filename	Trajectory file
--minrange aa	(opt.) First amino acid for decomposition
--maxrange aa	(opt.) Last amino acid for decomposition
--mpi np	(opt.) Number of threads for minimization

REBELOT in multiple frame mode generates some output files:

- Directories FRAME_xx, that contains the analysis of every frame, and in particular the original matrices M_{ij} in the file ele-vdw.dat and the reconstructed structures in snapshot.AMBER.pdb.

- `ele-vdw_average.dat` which contains the average matrix $\langle M_{ij} \rangle$
- `EIGENVAL.txt` and `EIGENVECT.txt`, which contain the eigenvalue and the eigenvectors from the diagonalization of $\langle M_{ij} \rangle$;
- `enematrix.dat`, which contains the reconstructed energy matrix $\tilde{M}_{ij}$ and its plot `enematrix.png`;
- `enedist.dat`, which contains the distribution of interaction energy vs. the residue position, and the plot `enedist.png`
- `REBELOT.log`, which contains useful informations about the run, and all the output given by any part of the workflow.

There are also other file (e.g. input files created for `sander`, `mm_pbsa.pl` and their output).

3.1.3 Cluster mode (-m c)

REBELOT can analyze clusters results. Having n representative clusters in a trajectory, REBELOT consider every frame obtaining n matrices M_{ij}. Having the population for each cluster, it computes the population-weighted average matrix $\langle M_{ij} \rangle$ and diagonalize it, obtaining the eigenvalues and eigenvectors that can be used to reconstruct the matrix $\tilde{M}_{ij}$. Obviously, the computation time scales with the number of frame contained in the trajectory.
In cluster mode, REBELOT works with the following syntax:

```
$ REBELOT.py -m c [-d] -f trajectory.pdb --cluster
      clusterfile [--minrange aa1 --maxrange aa2]
      [--mpi np]
```

where the options refer to

Option	Description
`-d`	Activate domain decomposition
`-f filename`	Trajectory file
`--cluster filename`	Cluster file, explained below
`--minrange aa`	(opt.) First amino acid for decomposition
`--maxrange aa`	(opt.) Last amino acid for decomposition
`--mpi np`	(opt.) Number of threads for minimization

The cluster file (`-c` option), has to be a single column text file containing the population for every frame.
REBELOT in cluster mode generates some output files:

- Directories FRAME_xx, that contains the analysis of every frame, and in particular the original matrices M_{ij} in the file ele-vdw.dat and the reconstructed structures in snapshot.AMBER.pdb.
- ele-vdw_average.dat which contains the weighted average matrix $\langle M_{ij} \rangle$
- EIGENVAL.txt and EIGENVECT.txt, which contain the eigenvalue and the eigenvectors from the diagonalization of $\langle M_{ij} \rangle$;
- enematrix.dat, which contains the reconstructed energy matrix $\tilde{M}_{ij}$ and its plot enematrix.png;
- enedist.dat, which contains the distribution of interaction energy vs. the residue position, and the plot enedist.png
- REBELOT.log, which contains useful informations about the run, and all the output given by any part of the workflow.

There are also other file (e.g. input files created for sander, mm_pbsa.pl and their output).

3.1.4 BEPPE mode (-m b)

REBELOT can perform an epitope search and representation (on a PyMOL-readable file [**?**]) using the pyBEPPE.py executable in the workflow, like in [**?**, **?**].
The workflow builds the approximated energy matrix $\tilde{M}_{ij}$ for a single structure (like in section 3.1.1) and then try to identify the residues which are in contact in a metric sense (i.e. under a threshold distance, fixed at 6 Åof beta carbons). Using the reference structure we build a contact matrix C_{ij} and a matrix of local coupling energy (MLCE) L_{ij} is computed in the following way

$$L_{ij} = C_{ij} \circ \tilde{M}_{ij}$$

where $\circ$ refers to the Hadamard product (or entrywise product).
At this point, the BEPPE algorithm selects the list of the amino acids with the minimal coupling energy to the rest of the protein residues. To define the uncoupled amino acids, we put a threshold on the percentage of the less energetic coupling on all the structure; this affect the sensitivity/specificity of the method.
In BEPPE mode, REBELOT works with the following syntax:

```
$ REBELOT.py -m b [-d] -f protein.pdb [-c threshold]
      [-t topology_file] [-M] [--matrix matrix.dat |
      --coevo coevo.dat]  [--mpi np]
```

where the options refer to

Option	Description
-d	Activate domain decomposition
-f filename	Structure file
--matrix filename	(opt.) Raw interaction matrix
--coevo filename	(opt.) Coevolutionary potential matrix
-c	(opt.) Threshold for the uncoupled residues (default: 15%)
-t	(opt.) Output file containing the contact map (default : topology.dat)
-M	(opt.) Output file containing the Matrix of the Local Coupling energy (mlce.dat)
--mpi np	(opt.) Number of threads for minimization

REBELOT in BEPPE mode generates some output files:

- `ele-vdw.dat` which contains the original matrix M_{ij} (if `--matrix` or `--coevo` options are not enabled)
- `EIGENVAL.txt` and `EIGENVECT.txt`, which contain the eigenvalue and the eigenvectors from the diagonalization of M_{ij};
- `enematrix.dat`, which contains the reconstructed energy matrix $\tilde{M}_{ij}$ and its plot `enematrix.png`;
- `snapshot.AMBER.pdb`, which is the structure parsed and possibly repaired by tleap;
- `beppe_snapshot.AMBER.pml` which contains the PyMOL layer with the patches;
- `REBELOT.log`, which contains useful informations about the run, and all the output given by any part of the workflow.

There are also other file (e.g. input files created for `sander`, `mm_pbsa.pl` and their output).

3.2 pyBEPPE.py

REBELOT contains also the BEPPE implementation in python, `pyBEPPE.py`. It is used in BEPPE mode (see previous section) and it can be used alone using the reconstructed matrix (or the eigenvalues/eigenvectors files) obtained with

REBELOT.py. It can be used to change the threshold value avoiding the structure minimization and the computation of GBSA matrix (which take 95% of the calculation time).
pyBEPPE.py works with the following syntax:

```
$ pyBEPPE.py -f protein.pdb  (-e energy_matrix |
      -v eigenvectors -a eigenvalues) [-s threshold]
      [-t topology_file] [-m]
```

where the options refer to

Option	Description
-f filename.pdb	Structure file
-e enermat	Reconstructed energy matrix file
-v eigenvec	Eigenvectors file
-a eigenval	Eigenvalues file
-s threshold	(opt.) Threshold for the uncoupled residues (default: 15%)
-t topology	(opt.) Output file containing the contact map (default : topology.dat)
-m	(opt.) Output file containing the Matrix of the Local Coupling Energies (mlce.dat)

pyBEPPE.py generates some output files:

- beppe_$filename.pml which contains the PyMOL layer with the patches, where $filename is the name of the structure file without the .pdb extension;
- beppe_$filename.log, which contains useful informations about the run, and all the output given by any part of the workflow. Also in this case $filename is the name of the structure file without the .pdb extension.
